# Real-Time Symptom Ratings Using Ecological Momentary Assessment Versus Traditional Questionnaires in Patients With Chronic Obstructive Pulmonary Disease: Observational Study

**DOI:** 10.2196/79001

**Published:** 2026-04-07

**Authors:** Banchia Palmen, Maarten van Herck, Yvonne M J Goërtz, Zjala Ebadi, Qichen Deng, Melissa S Y Thong, Chris Burtin, Jeannette B Peters, Roy T M Sprooten, Erik W M A Bischoff, Emiel F M Wouters, Jan H Vercoulen, Sarah Houben-Wilke, Daisy J A Janssen, Martijn A Spruit, Anouk W Vaes

**Affiliations:** 1 Department of Research and Development, Centre of Expertise for Chronic Organ Failure (CIRO), Hornerheide 1, Horn, 6085 NM, The Netherlands, 31 0475 587600; 2 Institute of Nutrition and Translational Research in Metabolism, Care and Public Health Research Institute, Faculty of Health, Medicine and Life Sciences, Maastricht University, Maastricht, The Netherlands; 3 Living Lab in Ageing and Long-Term Care, Maastricht University, Maastricht, The Netherlands; 4 Department of Health Services Research, Care and Public Health Research Institute, Faculty of Health, Medicine and Life Sciences, Maastricht University, Maastricht, The Netherlands; 5 Department of Medical Psychology, Radboud University Nijmegen, Nijmegen, The Netherlands; 6 Department of Pulmonary Diseases, Radboud University Medical Centre, Nijmegen, The Netherlands; 7 Medical Psychology, Amsterdam UMC Location University of Amsterdam, Amsterdam, The Netherlands; 8 Cancer Survivorship Outcomes and Epidemiology, German Cancer Research Center, Heidelberg, Germany; 9 REVAL—Rehabilitation Research Center, BIOMED—Biomedical Research Institute, Faculty of Rehabilitation Sciences, Hasselt University, Hasselt University, Diepenbeek, Belgium; 10 Department of Respiratory Medicine, Maastricht University Medical Centre, Maastricht, The Netherlands; 11 Department of General Practice, Erasmus University Medical Centre, Rotterdam, The Netherlands; 12 Department of Primary and Community Care, Radboud University Medical Center, Nijmegen, The Netherlands; 13 Department Internal Medicine, Sigmund Freud University Vienna, Vienna, Austria; 14 Department of Family Medicine, Care and Public Health Research Institute, Faculty of Health, Medicine and Life Sciences, Maastricht University, Maastricht, The Netherlands; 15 Department of Respiratory Medicine, Institute of Nutrition and Translational Research in Metabolism, Faculty of Health, Medicine and Life Sciences, Maastricht University, Maastricht, The Netherlands

**Keywords:** chronic obstructive pulmonary disease, COPD, ecological momentary assessment, questionnaires, symptom assessment

## Abstract

**Background:**

Questionnaires assessing symptoms in chronic obstructive pulmonary disease (COPD) rely on retrospective reporting, introducing recall bias and missing symptom variability. Ecological momentary assessment (EMA) addresses these limitations by capturing real-time symptom data.

**Objective:**

The aim of this study was to compare EMA symptom ratings with traditional questionnaires in patients with COPD.

**Methods:**

A subsample from the FAntasTIGUE study rated symptoms using questionnaires (visual analog scale, modified Medical Research Council Dyspnea Scale, Physical Activity Rating Scale-Dyspnea Questionnaire, Checklist Individual Strength-subscale Subjective Fatigue, Hospital Anxiety and Depression Scale, and COPD Assessment Test) and EMA. Participants rated breathlessness, fatigue, anxiety, and energy level on a 7-point Likert scale during 8 random EMA prompts daily for 5 days.

**Results:**

Among 54 participants (mean age 67, SD 7 y; forced expiratory volume in 1 s 53%, SD 20% predicted; n=35, 65% men), the EMA response rate was 76.1%. EMA scores were moderately to strongly correlated with questionnaire scores (*r*_s_=0.49-0.78, all *P*<.05) and varied across severity groups (all *P*<.05). EMA captured significant intraday and interday symptom variability.

**Conclusions:**

EMA is a valid tool for COPD symptom assessment, capturing real-time fluctuations to improve symptom management.

## Introduction

Chronic obstructive pulmonary disease (COPD) is a heterogeneous chronic lung condition characterized by persistent airflow limitation and respiratory symptoms such as breathlessness and fatigue [1,2]. Additionally, patients with COPD often experience psychological symptoms like anxious and depressed mood and lack of energy, which can substantially affect their health-related quality of life and functional status [3,4]. Despite this, breathlessness, fatigue, anxiety, and lack of energy are frequently underreported [5]. In both daily clinical practice and research, these symptoms are assessed using questionnaires, such as the modified Medical Research Council (mMRC) Dyspnea Scale, Checklist Individual Strength-subscale Subjective Fatigue (CIS-Fatigue), and Hospital Anxiety and Depression Scale (HADS) [6,7]. However, these traditional tools rely on retrospective self-reports, which may be limited by recall bias and fail to fully capture the expected dynamic nature of symptoms both intraday and interday [1,8-13].

Ecological momentary assessment (EMA) is a real-time data collection approach that seeks to overcome these limitations by facilitating data capture in patients’ natural environment [1,8,9,14]. By prompting patients to report breathlessness, fatigue, anxiety, and lack of energy at repeated intervals throughout the day, EMA minimizes memory-related biases and provides a more detailed depiction of symptom variability, offering a comprehensive view of daily symptom burden in patients with COPD. Despite the growing use of EMA in chronic respiratory disease research, there remains a limited understanding of how well EMA-based symptom ratings align with traditional questionnaires [9,15,16]. Establishing this relationship is essential to evaluate the measurement properties of EMA, specifically its convergent validity with questionnaires. By exploring to which extent EMA-derived symptom ratings correlate with well-established validated questionnaire scores, it can be determined whether EMA can serve as a valid complementary tool for symptom assessment in COPD.

This study aims to assess the convergent validity of EMA symptom ratings by exploring the associations between EMA-based and questionnaire-based measurements of breathlessness, fatigue, anxiety, and energy in patients with COPD. We seek to determine whether EMA provides unique insights into daily symptom fluctuations in COPD by examining moment-to-moment variability of symptoms both intraday and interday, which cannot be captured by traditional retrospective questionnaires. Our findings may have implications for improving symptom monitoring and management in daily clinical practice and research. By identifying moment-to-moment fluctuations in symptoms such as breathlessness, fatigue, anxiety, and lack of energy, EMA may enable timely and personalized adjustments in treatment or self-management strategies, potentially leading to more responsive and individualized care for patients with COPD. It is hypothesized that EMA symptom ratings would show significant positive correlations with traditional questionnaire scores, supporting convergent validity. Furthermore, we expect EMA to reveal substantial intraday and interday variability in breathlessness, fatigue, anxiety, and lack of energy that is not captured by retrospective questionnaires.

## Methods

### Design

This research was conducted as part of a multicenter, longitudinal, observational study in patients with COPD investigating physical, systemic, psychological, and behavioral factors associated with fatigue (FAntasTIGUE study) [17]. The prevalence of fatigue in patients with COPD was previously reported, as well as the association between physical activity, sedentarism, and EMA-derived symptom scores [3,18,19].

### Ethical Considerations

The study received approval from the Medical Research Ethics Committee United, Nieuwegein, The Netherlands (R17.036/NL60484.100.17), and written informed consent was obtained from all participants. Participation was entirely voluntary, and participants could withdraw at any time without consequence. Participants did not receive any financial compensation. Data were deidentified before analysis.

### Recruitment

Patients were recruited between 2018 and 2021 at outpatient clinics of the Department of Respiratory Medicine in Maastricht University Medical Center and the Department of Pulmonary Diseases of the Radboud University Medical Center in Nijmegen, as well as via general practitioners (Research Network Family Medicine Maastricht [20] and the Academic General Practitioner Network in the Nijmegen region). Additionally, patients with COPD who attended a peer support group meeting in Maastricht or Nijmegen, as well as patients recruited from primary and secondary care who previously participated in the Chance Study (NTR3416) and had consented to follow-up research, were invited to take part in the FAntasTIGUE study. To participate in the EMA substudy, patients had to meet the following inclusion criteria:

1. A diagnosis of COPD according to the Global Strategy for the Diagnosis, Management and Prevention of COPD (Global Initiative for Chronic Obstructive Lung Disease, grade 1A–4D) [2].
2. No exacerbation-related hospitalization and/or use of oral corticosteroids and/or antibiotics less than 4 weeks preceding enrollment.
3. Provided written informed consent.
4. Able to use an iPod.

Patients lacking sufficient understanding of the Dutch language and/or participating in concurrent intervention studies were excluded.

### Measurements

During baseline assessment, sociodemographic, physical, psychological, and behavioral characteristics were assessed as described elsewhere [17].

### Questionnaires

Breathlessness was assessed using 3 methods: mMRC Dyspnea Scale, item 1 of the Physical Activity Rating Scale-Dyspnea Questionnaire (PARS-D), and visual analog scale (VAS). The mMRC Dyspnea Scale assesses the current severity of activity-related dyspnea on a scale ranging from 0 (“only gets breathless with strenuous exercise”) to 4 (“too breathless to leave the house or breathless when dressing”) [21]. An mMRC score of 2 or higher is used as a threshold for separating “less breathlessness” from “more breathlessness” [2]. On item 1 of the PARS-D, patients had to rate the level of breathlessness on most days of the previous month from 1 (“not at all breathless”) to 10 (“very breathless”) [22]. Patients rated their level of breathlessness over the past 2 weeks using a VAS, which ranged from 0 (“not”) to 100 mm (“worst possible”) [23,24].

Fatigue was assessed using 2 methods: CIS-Fatigue and VAS. The CIS-Fatigue contains questions about fatigue-related complaints in the previous 2 weeks. For these analyses, we used both the score from item 1 and the CIS-Fatigue total score. Item 1 asks the patient if they are tired, ranging from 1 (“yes, that’s true”) to 7 (“no, that’s not true”). A total score of 26 points or lower on the CIS-Fatigue indicates normal fatigue, between 27 and 35 reflects mild fatigue, and 36 points or higher signifies severe fatigue [6]. Patients rated their level of fatigue over the past 2 weeks using the VAS, which ranged from 0 (“not”) to 100 mm (“worst possible”) [23,24].

Symptoms of anxiety and depression were measured using 2 methods: HADS and VAS. The HADS is divided into an anxiety and a depression subscale. Each subscale consists of 7 items and ranges from 0 to 21 points. A higher score on these subscales indicates a higher level of symptoms of anxiety or depression, respectively. A subscale score of 8 points or higher indicates possible symptoms of anxiety or depression, while a score of 11 points or higher indicates probable symptoms of anxiety or depression [7]. Patients rated their level of anxiety over the past 2 weeks using the VAS, which ranged from 0 (“not”) to 100 mm (“worst possible”) [23,24].

Energy was assessed with item 8 of the COPD Assessment Test (CAT), which assesses the current energy level on a scale from 0 to 5, with a higher score indicating less energy [25]. All questionnaires used in this study are well-validated and widely applied instruments for assessing symptoms in patients with COPD [2,26,27]. However, for some analyses, single items from these questionnaires were used to allow direct comparison with specific EMA items reflecting corresponding symptom dimensions.

### Ecological Momentary Assessment

Electronic EMA questionnaires were administered over 5 consecutive days using an Apple iPod Touch with a custom-installed app PsyMate (version 1.1b). If possible, the EMA assessments began the day immediately following the baseline assessment. Patients were instructed to respond to each questionnaire as soon as possible upon hearing the beep and to keep their usual day and night routine. If patients did not respond within 15 minutes after the beep, the questionnaire was skipped. PsyMate generated a beep at 8 random times each day between 7:30 AM and 10:30 PM. Patients had to rate 10 symptoms (“I feel: relaxed; short of breath; energetic; cheerful; insecure; irritated; satisfied; anxious; tired; and mentally fit”) with a 7-point Likert scale ranging from 1 (“Not”) to 7 (“Very”). For these analyses, 4 symptoms were included: breathlessness, fatigue, anxiety, and energy. Patients who completed less than one-third of the total beeps (ie, <13 out of 40 beeps) were excluded [28]. An EMA questionnaire completion time of 10 minutes or longer was considered invalid, and this EMA data point was excluded.

### Data Analyses

All analyses were performed using SPSS (version 28.0.1.1) and R statistical software (version 4.4.2). The distribution of data was tested using the Shapiro-Wilk test with a 2-tailed significance of less than .05. Data were reported as mean (SD), median (IQR), percentages, and numbers, where appropriate. In the analyses, EMA item “I feel short of breath” was compared with mMRC Dyspnea Scale score, PARS-D score, and VAS breathlessness score. EMA item “I feel tired” was compared with CIS-Fatigue item 1 score, CIS-Fatigue total score, and VAS Fatigue score. EMA item “I feel anxious” was compared with HADS anxiety score and VAS anxiety score, while EMA item “I feel energetic” was compared with CAT item 8 score. To evaluate the convergent validity of EMA measures, associations between EMA symptom ratings and corresponding questionnaire scores were examined using Spearman correlation coefficients and multilevel linear mixed models (MLMs).

Median EMA scores for all patients were calculated. Spearman correlation analyses were performed between median EMA scores and corresponding questionnaire scores [29]. Subsequently, MLMs were applied to analyze all EMA data points. In these models, EMA scores were treated as dependent variables, while questionnaire scores were included as fixed effects. Patient ID was specified as a random intercept to account for the correlation of intraindividual repeated observations. MLMs were performed using the “mixed” command, restricted maximum likelihood estimations, and a significance of less than .05. Mann-Whitney *U* tests were used to assess differences in median EMA scores across symptom severity groups based on established cutoff values of the mMRC Dyspnea Scale (0‐1/≥2), CIS-Fatigue (8‐35/≥36), and HADS questionnaire (1‐7/≥8). Patients were categorized into the symptom severity groups based on their corresponding questionnaire scores.

To assess intraday and interday symptom variability, we used MLMs with EMA symptom scores as the dependent variable. For each symptom (breathlessness, fatigue, anxiety, and energy), time of day (expressed as hours since 8:00 AM) and day number (days 1‐5) were included as fixed effects to model temporal trends. Random intercepts for each participant accounted for interindividual differences in baseline symptom levels, while random slopes for time of day allowed the trajectory of symptoms to change throughout the day to vary interindividuals. Variance components were extracted from the MLMs to estimate the proportion of total variance attributable to intraindividual fluctuations and interindividual differences. Intraclass correlation coefficients (ICC) were calculated as the ratio of interindividual variance to total variance after accounting for time of day and day effects. ICC values close to 1 indicate that most variability is explained by differences between individuals, whereas lower ICC values indicate greater intraindividual variability.

## Results

### Overview

Sixty patients were assessed for eligibility. Six patients were excluded because of no available EMA data (n=2), invalid EMA data (<13 beeps in total; n=3), or incorrect loading of the EMA data (n=1). Consequently, 54 patients were included in the current analysis.

### Participants

The majority of patients were men (35/54, 65%) with an average age of 67 years (Table 1). Most patients had moderate-to-severe COPD (49/54, 91%), and almost half had at least one exacerbation in the previous 12 months (25/54, 46%). Across the 9 questionnaires that were used in the analysis, the overall response rate was high (471/486, 97%). Severe dyspnea was reported by 39% (21/52) and 43% (23/53) experienced severe fatigue. In addition, 9% (5/52) and 11% (6/52) of the patients had symptoms of anxiety and depression, respectively. The EMA subsample included in the current analyses is highly comparable to the overall FAntasTIGUE cohort in terms of key demographic and clinical characteristics. Results of the full FAntasTIGUE cohort have recently been published [19].

**Table 1.** Baseline characteristics.

Variable^a^	N	Value
Age (y), mean (SD)	54	67 (7)
Male, n (%)	54	35 (65)
Married/living together, n (%)	53	35 (65)
Currently employed, n (%)	52	7 (13)
Current smoker, n (%)	54	9 (17)
CCI^b^ (points), median (IQR)	54	1 (1-2)
Exacerbation in previous 12 mo (0), n (%)	54	29 (54)
Exacerbation in previous 12 mo (1), n (%)	54	9 (17)
Exacerbation in previous 12 mo (≥2), n (%)	54	16 (30)
Exacerbation-related hospitalization in previous 12 mo (0), n (%)	54	45 (83)
Exacerbation-related hospitalization in previous 12 mo (1), n (%)	54	6 (13)
Exacerbation-related hospitalization in previous 12 mo (≥2), n (%)	54	3 (6)
BMI (kg/m^2^), median (IQR)	54	25 (23‐30)
FEV_1_^c^ (% predicted), mean (SD)	54	53 (20)
GOLD^d^ I/II/III/IV, n (%)	54	5 (9); 22 (41); 22 (41); 5 (9)
FEV_1_/FVC^e^ (%), median (IQR)	54	46 (35‐57)
mMRC^f^ Dyspnea (grade), median (IQR)	52	1 (0‐2)
mMRC^f^ ≥2, n (%)	52	21 (39)
PARS-D^g^ item 1 (Shortness of breath) (score), median (IQR)	54	5 (2-6)
VAS^h^ breathlessness (score), median (IQR)	52	25 (10‐62)
CIS-Fatigue^i^ item 1 (I feel tired) (score), median (IQR)	53	4 (2-7)
CIS-Fatigue^i^ (points), median (IQR)	53	33 (21‐49)
Severe fatigue (≥36), n (%)	53	23 (43)
VAS^h^ fatigue (score), median (IQR)	52	40 (13‐60)
HADS^j^-Anxiety (points), median (IQR)	52	4 (1-8)
Symptoms of anxiety (8‐10 points), n (%)	52	9 (17)
Symptoms of anxiety (≥11 points), n (%)	52	5 (9)
HADS^j^-Depression (points), median (IQR)	52	4 (1-8)
Symptoms of depression (8‐10 points), n (%)	52	7 (13)
Symptoms of depression (≥11 points), n (%)	52	6 (11)
VAS^h^ anxiety (score), median (IQR)	52	2 (1-15)
CAT^k^ item 8 (I have a lot of energy) (score), median (IQR)	52	3 (2-4)

a Quantitative variables are presented as mean (SD) or median (IQR) for skewed variables.

b CCI: Charlson Comorbidity Index.

c FEV1 % predicted: Forced expiratory volume in 1 second as percentage from the predicted volume.

d GOLD: Global Initiative for Chronic Obstructive Lung Disease.

e FEV1/FVC %: the forced expiratory volume in 1 second to forced vital capacity ratio.

f mMRC: modified Medical Research Council Dyspnea Scale.

g PARS-D: Physical Activity Rating Scale-Dyspnea questionnaire.

h VAS: visual analog scale.

i CIS-Fatigue: Checklist Individual Strength-subscale Subjective fatigue.

j HADS: Hospital Anxiety and Depression Scale.

k CAT: COPD Assessment Test.

### Ecological Momentary Assessment

**Figure 1.**
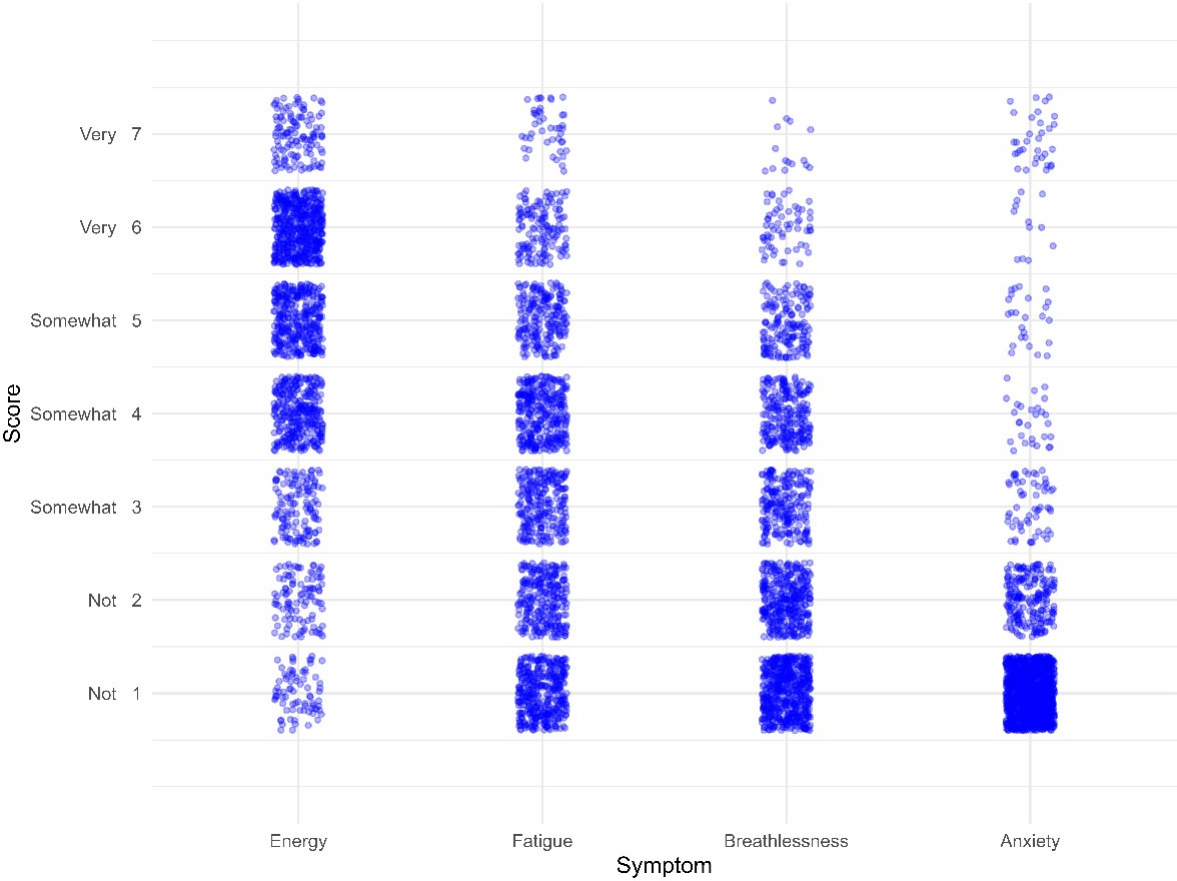
Jittered scatterplot of the ecological momentary assessment–based symptoms scored on a 7-point Likert scale for 5 consecutive days.

One patient completed 4 days of EMA measurements, and 53 patients completed 5 days. Of the 2160 maximal possible EMA responses (54 participants × 5 days × 8 prompts per day), 1644 EMA responses were valid and could be analyzed (response rate: 76.1%). Median (IQR) time of completing the EMA questionnaire was 1 minute and 2 seconds (00:49–01:21). During the 5 days, patients generally rated feeling somewhat energetic (median 5 points, IQR 2), somewhat tired (median 3 points, IQR 2), not short of breath (median 2 points, IQR 3), and not anxious (median 1 point, IQR 0). Nevertheless, a large variation across the 7-point Likert scale was observed between patients (Figure 1). In Multimedia Appendix 1, the median EMA scores and total number of EMA responses per patient are shown.

### Association

**Figure 2.**
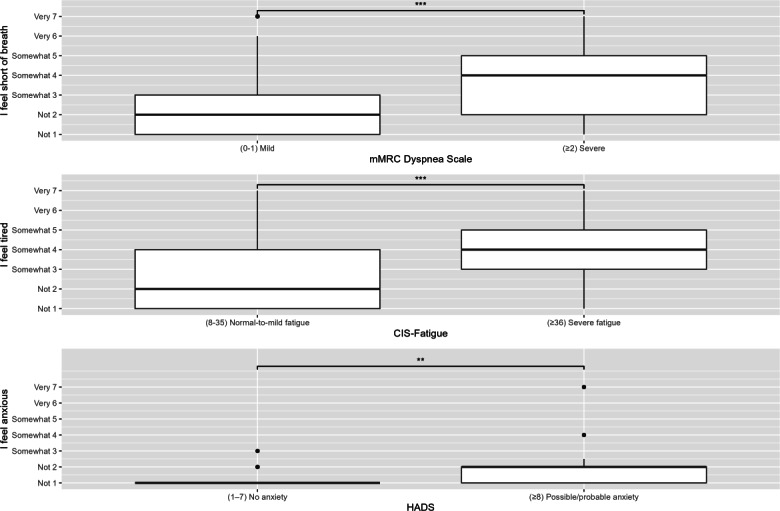
Ecological momentary assessment (EMA) breathlessness scores divided by the modified Medical Research Council (mMRC) Dyspnea cutoff values. EMA tiredness scores divided by Checklist Individual Strength-subscale Subjective Fatigue (CIS-Fatigue) cutoff values. EMA anxious scores divided by the Hospital Anxiety and Depression Scale (HADS) cutoff values.

Median EMA scores and corresponding questionnaires showed moderate-to-strong correlations (Table 2). Patients with an mMRC grade of 0 to 1 reported significantly lower EMA scores for “I feel short of breath” compared to those with an mMRC grade of 2 or higher (*P*<.001; Figure 2). Similarly, patients with normal-to-mild CIS-Fatigue total scores reported significantly lower EMA scores for “I feel tired” compared to those with severe CIS-Fatigue scores (*P*<.001). Patients with no symptoms of anxiety had significantly lower EMA scores for “I feel anxious” compared to those with possible or probable anxiety (*P*=.01).

**Table 2.** Correlations between median ecological momentary assessment scores and corresponding questionnaires.

EMA^a^ item and questionnaire		Spearman correlation coefficient^b^
I feel short of breath		
	mMRC^c^ Dyspnea Scale	0.63
	PARS-D^d^ item 1 Shortness of breath	0.76
	VAS^e^ breathlessness	0.74
I feel tired		
	CIS-Fatigue^f^ item 1 (I feel tired)	0.74
	CIS-Fatigue^f^ total score	0.76
	VAS^e^ fatigue	0.78
I feel anxious		
	HADS^g^-Anxiety	0.55
	VAS^e^ anxiety	0.49
I feel energetic		
	CAT^h^ Item 8 (I have a lot of energy)	−0.65

a EMA: ecological momentary assessment.

b *P*<.001.

c mMRC: modified Medical Research Council Dyspnea Scale.

d PARS-D: Physical Activity Rating Scale-Dyspnea questionnaire.

e VAS: visual analog scale.

f CIS-Fatigue: Checklist Individual Strength-subscale Subjective Fatigue.

g HADS: Hospital Anxiety and Depression Scale.

h CAT: COPD Assessment Test.

Multilevel models revealed that higher scores on the mMRC Dyspnea Scale, PARS-D item 1, and VAS breathlessness were associated with higher EMA scores for “I feel short of breath” (Table 3). Specifically, a one-unit increase in mMRC Dyspnea Scale score corresponded to a 0.6-point increase in EMA score. For example, a patient with an mMRC score of 1 (the median in our sample) would be expected to report an average EMA breathlessness score of approximately 3.1 on the 7-point scale. Corresponding expected EMA scores for each questionnaire value are provided in Multimedia Appendix 2. Similarly, higher scores on CIS-Fatigue item 1, CIS-Fatigue total score, and VAS Fatigue were associated with higher EMA scores for “I feel tired.” Elevated HADS-Anxiety and VAS anxiety scores were also associated with higher EMA scores for “I feel anxious.” Conversely, higher scores on CAT item 8 were associated with lower EMA scores for “I feel energetic.”

**Table 3.** Associations between symptoms and corresponding questionnaires.

EMA^a^ item and questionnaire (range)		Estimate (SE)^b^
I feel short of breath		
	mMRC^c^ Dyspnea Scale (0-4)	0.600 (0.126)
	PARS-D^d^ Item 1 Shortness of breath (1-10)	0.399 (0.046)
	VAS^e^ breathlessness (0‐100)	0.035 (0.004)
I feel tired		
	CIS-Fatigue^f^ item 1 (I feel tired) (1-7)	0.438 (0.053)
	CIS-Fatigue^f^ total score (8-56)	0.071 (0.009)
	VAS^e^ Fatigue (0‐100)	0.037 (0.005)
I feel anxious		
	HADS^g^-Anxiety (0-21)	0.166 (0.022)
	VAS^e^ anxiety (0‐100)	0.035 (0.004)
I feel energetic		
	CAT^h^ Item 8 (I have a lot of energy) (0-5)	−0.602 (0.099)

a EMA: ecological momentary assessment.

b *P*<.001.

c mMRC: Modified Medical Research Council Dyspnea Scale.

d PARS-D: Physical Activity Rating Scale-Dyspnea Questionnaire.

e VAS: visual analog scale.

f CIS-Fatigue: Checklist Individual Strength-subscale Subjective Fatigue.

g HADS: Hospital Anxiety and Depression Scale.

h CAT: COPD Assessment Test.

### Variability

The ICC for EMA “I feel short of breath” was 0.624, indicating that 62.4% of the variance in EMA scores was explained by interindividual differences, while 37.6% was attributable to intraindividual fluctuations over time. Time of day (expressed as hours since 8:00 AM) had a significant negative association with EMA breathlessness ratings (*β*=−1.3×10^−2^, *P*=.03; Table 4), with scores being highest in the morning and declining throughout the day. Additionally, scores on day 2 were significantly lower than day 1 (*β*=−1.6×10^−1^, *P*=.03), whereas differences for subsequent days were not statistically significant.

**Table 4.** Intraclass correlation coefficient (ICC) and associations between the ecological momentary assessment (EMA) symptoms, time of day, and day number, with the first day being the reference day.

EMA item^a^	ICC^b^	Estimate (SE)				
		Time of day^c^	Day 2^d^	Day 3^d^	Day 4^d^	Day 5^d^
I feel short of breath	0.624	−1.3×10^−2^ (5.8×10^-3^)^e^	−1.6×10^−1^ (7.8×10^-2^)^e^	−1.4×10^−1^ (7.8×10^-2^)	−9.7×10^−2^ (7.9×10^-2^)	−1.3×10^−1^ (7.9×10^-2^)
I feel tired	0.584	3.7×10^−2^ (1.1×10^-2^)^f^	8.9×10^−3^ (9.0×10^-2^)	−8.1×10^−3^ (9.1×10^-2^)	4.4×10^−2^ (9.2×10^-2^)	−2.1×10^−2^ (9.2×10^-2^)
I feel anxious	0.683	5.2×10^−4^ (3.9×10^-3^)	−1.2×10^−1^ (5.4×10^-2^)^e^	−8.6×10^−2^ (5.4 × 10^-2^)	−1.1×10^−1^ (5.5×10^-2^)	−1.0×10^−1^ (5.5×10^-2^)
I feel energetic	0.605	−1.6×10^−2^ (9.1×10^-3^)	7.6×10^−2^ (7.7×10^-2^)	6.0×10^−2^ (7.7×10^-2^)	9.4×10^−3^ (7.8×10^-2^)	1.8×10^−2^ (7.8×10^-2^)

a EMA: ecological momentary assessment.

b The ICC represents the proportion of total variance in EMA scores attributable to interindividual differences, after accounting for temporal effects.

c Estimates (β) for time of day indicate the change in symptom rating per hour since 8:00 AM. Positive β values indicate increasing symptom ratings per hour and negative β values indicate decreasing ratings. SEs are provided in parentheses.

d Estimates for days 2-5 represent the difference in symptom ratings compared to the first day (reference).

e *P*<.05.

f *P*<.01.

**Figure 3.**
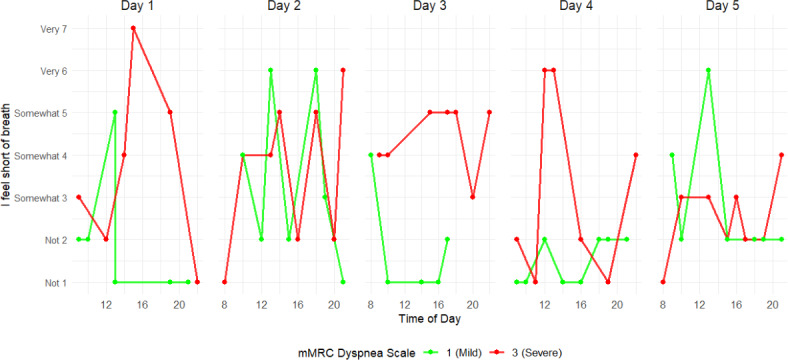
Five consecutive days of ecological momentary assessment breathlessness scores of 2 patients from different modified Medical Research Council (mMRC) Dyspnea subgroups. Each point represents a momentary breathlessness rating (1=“not at all breathless” to 7=“very breathless”) collected across 8 random prompts per day. The green line is of a patient with low breathlessness severity according to the mMRC Dyspnea Scale, and the red line is of a patient with high breathlessness severity.

Figure 3 illustrates EMA data for “I feel short of breath” for 2 patients with low and high mMRC grades. The figure demonstrates substantial intraday and interday variability in scores for both patients, showing that low and high EMA scores occurred regardless of the mMRC dyspnea grade.

**Figure 4.**
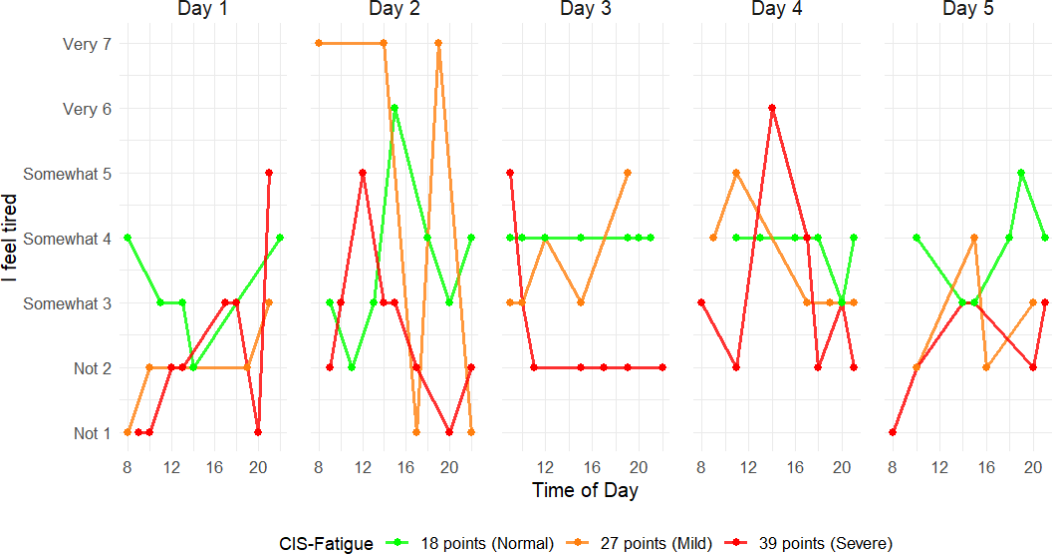
Five consecutive days of ecological momentary assessment fatigue scores of three patients from different Checklist Individual Strength-subscale Subjective Fatigue (CIS-Fatigue) subgroups. Each point represents a momentary tiredness rating (1=“not at all tired” to 7=“very tired”) collected across 8 random prompts per day. The green line is of a patient with normal fatigue according to the CIS-Fatigue, the orange line is of a patient with mild fatigue, and the red line is of a patient with severe fatigue.

For EMA “I feel tired,” the ICC was 0.584. Time of day had a significant effect on EMA scores (*β*=3.7×10^-2^, *P*=.002), where scores were lowest in the morning (≈8:00 AM) and increased during the day. Day number had no significant effect. Figure 4 depicts daily intraday variations in EMA scores for patients in both normal, mild, and severe CIS-Fatigue groups.

**Figure 5.**
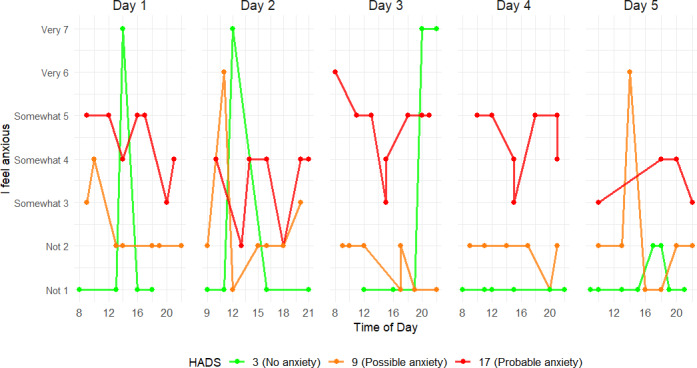
Five consecutive days of ecological momentary assessment anxiety scores of 3 patients from different Hospital Anxiety and Depression Scale (HADS) subgroups. Each point represents a momentary anxious rating (1=“not at all anxious” to 7=“very anxious”) collected across 8 random prompts per day. The green line is a patient with no anxiety symptoms according to the HADS, the orange line is a patient with possible symptoms of anxiety, and the red line is a patient with probable symptoms of anxiety.

ICC for EMA “I feel anxious” showed the highest interindividual variability, which was 0.683. Day 2 scores were significantly lower than day 1 (*β*=−1.2×10^-1^, *P*=.02), but there was no significant effect of time of day. Figure 5 highlights intraday fluctuations in EMA scores across all patient groups, irrespective of their HADS subgroup.

For EMA “I feel energetic,” the ICC was 0.605. Neither time of day nor day number showed significant effects in the model.

## Discussion

### Principal Findings

This study aimed to assess convergent validity of EMA symptom ratings by exploring associations between measuring symptoms with established questionnaires and EMA in patients with COPD. Moderate-to-strong correlations and significant associations support the convergent validity of EMA symptom ratings, with EMA effectively differentiating symptom severity and capturing intraday and interday variability which is present in the current study population.

Questionnaires provide a retrospective single-point observation that summarizes symptoms experienced over the previous weeks or months, whereas EMA collects real-time data in patients’ natural environment, minimizing recall bias [8]. While questionnaires may rely on patients’ general recollection of symptom burden, EMA focuses on the immediate context in which symptoms are experienced, offering a complementary perspective. Our findings indicate that both assessment tools measure symptoms accordingly; however, EMA can measure variability. Interestingly, the moderate-to-strong correlations observed in our study align with findings from previous studies conducted in different populations, which reported similar relationships between EMA and retrospective self-report measures in assessing clinical symptoms and behaviors [30-33]. This consistency across studies suggests that, despite their methodological differences, both approaches approximate the same symptom burden over time. However, momentary assessments using EMA may also capture subtle fluctuations influenced by contextual factors, which retrospective self-reports may overlook [34].

The substantial interday and intraday variability observed in EMA scores for breathlessness, fatigue, and anxiety highlights a key advantage of EMA over traditional assessments [16]. This emphasizes that traditional self-report measures often fail to capture such variability due to recall biases and the aggregation of experiences over extended timeframes. In the study of Solhan et al [16], EMA indices only showed modest agreement with retrospective reports of affective instability, further underscoring the limitations of relying solely on retrospective assessment. Similarly, our findings showed that patients with low mMRC, CIS-Fatigue, and HADS scores sometimes reported high EMA scores of the corresponding symptoms, illustrating the heterogeneity of symptoms intraindividual and across different time points [10].

### Clinical Practice and Research

EMA’s ability to capture real-time symptom variability provides clinicians with a richer and more nuanced dataset to inform personalized treatment plans. For example, when patients report a consistent pattern of increasing fatigue through EMA, this could lead to targeted behavioral strategies such as activity pacing (eg, scheduling lower-demand activities during that period) and timing of short rest breaks. Similarly, if EMA reveals increased feelings of anxiety around specific situations (eg, during exertion, social activity, or waking up), then this could trigger timely psychological-support interventions such as brief monitored relaxation techniques, guided breathing exercises, or preemptive alert via a mobile app to initiate mindfulness at forecasted high-risk times. In pulmonary rehabilitation, incorporating EMA can help patients gain insight into how daily lifestyle behaviors trigger certain symptoms and identify windows of vulnerability when symptoms tend to worsen. By visualizing the intraday and interday symptom fluctuations, clinicians and patients can co-develop personalized coping strategies such as adjusting nonpharmacological tactics (eg, paced walking, energy-conserving breathing techniques) during windows of higher symptom burden to remind patients to apply these tactics when indicated. EMA has been shown to enhance the detection of treatment effects, as evidenced in studies where EMA demonstrated superior sensitivity to change and convergence with structured clinical assessments [9,35]. By incorporating EMA alongside traditional measures, researchers can achieve a more comprehensive understanding of symptom variability and treatment efficacy. A potential drawback is that patient burden for completing EMA could be high, particularly for patients who are not very technically proficient or not stable [16].

### Methodological Considerations and Limitations

Both assessment tools demonstrated high response rates. While the EMA response rate in this study was comparable to previous research, adherence remains a critical factor for its successful implementation. Strategies to improve compliance, such as financial incentives or tailored EMA schedules, could enhance data quality [9]. Additionally, exclusion of patients with insufficient EMA data underscores the importance of establishing minimum response thresholds to ensure data reliability. Another limitation is that the self-reported symptoms may still be influenced by momentary factors such as mood, context, or tasks, even with EMA. The integration of EMA with objective measures, such as actigraphy or physiological monitoring, could provide a better understanding of how symptoms relate to specific activities and daily behaviors. This approach gives a more nuanced and objective assessment of symptom burden, capturing the dynamic interplay between symptoms and activity levels and potentially enhancing the validity of findings [15]. The median EMA score was used to summarize symptom burden across the 5-day period, which reduces the influence of extreme values and aligns with questionnaire measures. However, this approach does not capture fluctuations at specific times of day, such as morning or evening peaks. Future studies could explore time-specific EMA scores to provide more detailed insight into diurnal symptom variability. Additionally, symptom experiences often differ between sexes, and the predominance of male participants in this study may have influenced the results [36,37]. Furthermore, selection bias should also be considered, as participants who are more technologically proficient or motivated may be more likely to adhere to EMA protocols [38]. This may limit generalizability, particularly among older adults or those with severe COPD who could find frequent digital reporting burdensome [39]. Future studies should explore adaptive EMA designs or simplified interfaces to minimize this barrier and broaden participation. Despite these limitations, convergent validity remained high, even though EMA assessments were conducted after patients completed the questionnaires. Patients may have experienced a better or worse week in terms of symptoms before or during the EMA assessment period. Notably, some questionnaires assess symptoms over longer time frames, such as the past month or two weeks, which may capture a different temporal perspective on symptom burden. Another important methodological aspect concerns potential recall bias inherent in retrospective questionnaires. Traditional self-report instruments require participants to summarize symptoms experienced over extended periods, which may lead to distortions due to memory limitations or recency effects [12,13]. EMA mitigates this limitation by collecting data in real time, reducing recall-related error and providing a more accurate temporal representation of symptom burden. Furthermore, this study used single items from validated questionnaires (eg, PARS-D item 1, CIS-Fatigue item 1, CAT item 8). Although these items originate from instruments with well-established psychometric properties, individual items may not fully represent the multidimensional constructs assessed by the complete questionnaires. Together, these findings underscore the complementary strengths of EMA to provide a more nuanced understanding of symptom variability while still aligning with traditional measures of symptom severity.

### Conclusions

This study demonstrates that EMA is a validated tool for symptom assessment in patients with COPD. EMA showed moderate-to-strong correlations and significant associations with established questionnaires while capturing both interday and intraday symptom variability. The ability to reflect real-time fluctuations provides a more detailed and dynamic understanding of daily symptom burden, underscoring EMA’s added value for enhancing symptom monitoring and informing personalized management in COPD.

## Supplementary material

10.2196/79001 Multimedia Appendix 1 Median ecological momentary assessment (EMA) symptom scores and the total number of EMA responses per patient over 5 consecutive days.

10.2196/79001 Multimedia Appendix 2 Minimum, median, and maximum EMA symptom scores derived from questionnaire scores using the specified formulas.

## Abbreviations:

CAT: Chronic Obstructive Pulmonary Disease Assessment Test
CIS-Fatigue: Checklist Individual Strength-subscale Subjective Fatigue
COPD: chronic obstructive pulmonary disease
EMA: ecological momentary assessment
HADS: Hospital Anxiety and Depression Scale
ICC: intraclass correlation coefficient
MLM: multilevel linear mixed model
mMRC: modified Medical Research Council
PARS-D: Physical Activity Rating Scale-Dyspnea questionnaire
VAS: visual analog scale
