# Supplementary material for: Real-Time Symptom Ratings Using Ecological Momentary Assessment Versus Traditional Questionnaires in Patients With Chronic Obstructive Pulmonary Disease: Observational Study
Source: JMIR Med Inform. 2026 Apr 7;14:e79001. doi: 10.2196/79001 (PMC13055937; doi:10.2196/79001)
Supplement: Multimedia Appendix 1 [file medinform-v14-e79001-s001.docx]

Table 1: Median ecological momentary assessment (EMA) symptom scores and the total number of EMA responses per patient over 5 consecutive days. Each patient could provide a maximum of 5 × 8 = 40 responses.

| Patient ID | Breathlessness | Tired | Anxious | Energetic | Total number of EMA responses |
| --- | --- | --- | --- | --- | --- |
| 1 | 4 | 4 | 1 | 4 | 16 |
| 2 | 3 | 7 | 2.5 | 4 | 18 |
| 3 | 4 | 4.5 | 2 | 4 | 20 |
| 4 | 3 | 6 | 2 | 4 | 21 |
| 5 | 1 | 3 | 1 | 5 | 23 |
| 6 | 2 | 3 | 1 | 4 | 23 |
| 7 | 3 | 4 | 3 | 4 | 23 |
| 8 | 1 | 3 | 1 | 5 | 24 |
| 9 | 2 | 3 | 1 | 3 | 24 |
| 10 | 1 | 1 | 1 | 6 | 24 |
| 11 | 4 | 4 | 2 | 3 | 25 |
| 12 | 1 | 1 | 1 | 5 | 25 |
| 13 | 4 | 4 | 1 | 4 | 26 |
| 14 | 4 | 4 | 1 | 5 | 27 |
| 15 | 6 | 6 | 7 | 1 | 27 |
| 16 | 3 | 3 | 1 | 6 | 27 |
| 17 | 1 | 1 | 1 | 6 | 28 |
| 18 | 5 | 5 | 2 | 4 | 28 |
| 19 | 3 | 3 | 1 | 6 | 29 |
| 20 | 1 | 4 | 1 | 4 | 30 |
| 21 | 4 | 4 | 1 | 3 | 30 |
| 22 | 1 | 2.5 | 1 | 5 | 30 |
| 23 | 3 | 2 | 1 | 5 | 31 |
| 24 | 5 | 5 | 1 | 2 | 31 |
| 25 | 1 | 2 | 1 | 6 | 31 |
| 26 | 1 | 3.5 | 1 | 6 | 31 |
| 27 | 1 | 3.5 | 1 | 5 | 31 |
| 28 | 2 | 2 | 2 | 6 | 31 |
| 29 | 4 | 4 | 1 | 4 | 32 |
| 30 | 3 | 5 | 1 | 5 | 32 |
| 31 | 1 | 1 | 1 | 5 | 32 |
| 32 | 2.5 | 3 | 1 | 6 | 32 |
| 33 | 2 | 3 | 1 | 5 | 33 |
| 34 | 3 | 3 | 2 | 5 | 33 |
| 35 | 4 | 5 | 4 | 4 | 33 |
| 36 | 2 | 4 | 1 | 5 | 33 |
| 37 | 1 | 2 | 1 | 6 | 33 |
| 38 | 1 | 1 | 1 | 6 | 33 |
| 39 | 1 | 1 | 1 | 5 | 34 |
| 40 | 2 | 2 | 2 | 4 | 34 |
| 41 | 3 | 3 | 1 | 1 | 35 |
| 42 | 2 | 4 | 1 | 4 | 35 |
| 43 | 4 | 4 | 1 | 4 | 35 |
| 44 | 1 | 1 | 1 | 6 | 35 |
| 45 | 3.5 | 1 | 1 | 6 | 36 |
| 46 | 1 | 1 | 1 | 7 | 36 |
| 47 | 5 | 4 | 1 | 6 | 37 |
| 48 | 2 | 2 | 2 | 5 | 37 |
| 49 | 2 | 3 | 1 | 7 | 38 |
| 50 | 2 | 6 | 2 | 3 | 38 |
| 51 | 2 | 2 | 1 | 6 | 38 |
| 52 | 2 | 2 | 1 | 6 | 38 |
| 53 | 4 | 5 | 1 | 4 | 38 |
| 54 | 1 | 1 | 1 | 6 | 40 |
