# Supplementary material for: Real-Time Symptom Ratings Using Ecological Momentary Assessment Versus Traditional Questionnaires in Patients With Chronic Obstructive Pulmonary Disease: Observational Study
Source: JMIR Med Inform. 2026 Apr 7;14:e79001. doi: 10.2196/79001 (PMC13055937; doi:10.2196/79001)
Supplement: Multimedia Appendix 2 [file medinform-v14-e79001-s002.docx]

Table 1: Minimum, median, and maximum EMA symptom scores derived from questionnaire scores using the specified formulas.

| EMA | Formula | Questionnaire | Minimum^a^ | Median^a^ | Maximum^a^ |
| --- | --- | --- | --- | --- | --- |
|  |  |  |  |  |  |
| I feel short of breath | 2.459 + 0.600 X | mMRC dyspnea scale^b^ | 0  2.5 | 1  3.1 | 4  4.9 |
|  | 0.868 + 0.399 X | PARS-D Item 1 Shortness of breath^c^ | 1  1.3 | 5  2.9 | 10  4.9 |
|  | 1.471 + 0.035 X | VAS breathlessness^d^ | 0  1.5 | 25  2.3 | 100  5 |
| I feel tired | 1.388 + 0.438 X | CIS-Fatigue item 1 (I feel tired)^e^ | 1  1.8 | 4  3.1 | 7  4.5 |
|  | 0.904 + 0.071 X | CIS-Fatigue total score^e^ | 8  1.5 | 33  3.2 | 56  4.9 |
|  | 1.886 + 0.037 X | VAS Fatigue^d^ | 0  1.9 | 40  3.4 | 100  5.6 |
| I feel anxious | 0.701 + 0.166 X | HADS – Anxiety^f^ | 0  0.7 | 4  1.4 | 21  4.2 |
|  | 1.072 + 0.035 X | VAS Anxiety^d^ | 0  1.1 | 2  1.1 | 100  4.6 |
| I feel energetic | 6.300 + -0.602 X | CAT Item 8 (I have a lot of energy)^g^ | 0  6.3 | 3  4.5 | 5  3.3 |

^aupper-line is the questionnaire score and the lower-line is the calculated EMA score using the formula, bmMRC: Modified Medical Research Council dyspnea scale, cPARS-D: Physical Activity Rating Scale - Dyspnea Questionnaire, dVAS: Visual Analogue Scale, eCIS-Fatigue: Checklist Individual Strength - subscale subjective fatigue, fHADS: Hospital Anxiety and Depression Scale, gCAT: COPD Assessment Test.^
